# Supplementary material for: HSeeker: an algorithm for systematic H-DNA sequence identification
Source: bioRxiv. 2026 Jul 16:2026.07.10.737678. Preprint. [Version 1] doi: 10.64898/2026.07.10.737678 (PMC13404959; doi:10.64898/2026.07.10.737678)
Supplement: Supplementary Figure 1: [file NIHPP2026.07.10.737678v1-supplement-1.pdf]

bioRxiv preprint doi: https://doi.org/10.64898/2026.07.10.737678; this version posted July 16, 2026. The copyright holder for this preprint (which was not certified by peer review) is the author/funder, who has granted bioRxiv a license to display the preprint in perpetuity. It is made available under a CC-BY 4.0 International license.

Supplementary Figures

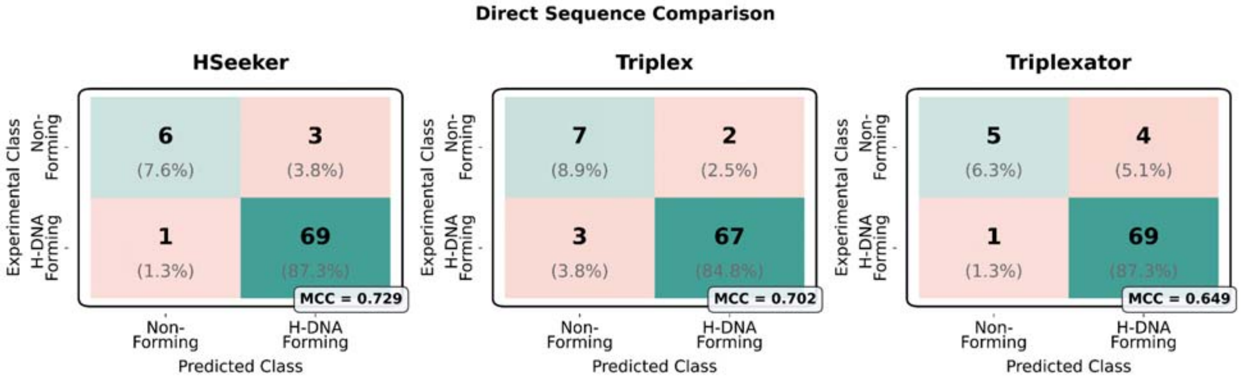

Supplementary Figure 1: Experimental H-DNA-forming (n=70) and non-forming (n=9) sequences were used to evaluate HSeeker classification performance. Direct sequence-level comparison of HSeeker, Triplex[21], and Triplexator[25] on 79 curated experimental sequences. Confusion matrices compare experimentally defined sequence classes with predicted classes for HSeeker, Triplex [21], and Triplexator [25] recovery of H-DNA-forming and non-forming sequences.
